# 

**Published:** 1887-12-17

**Authors:** 


PRICE ONE PENNY.

No. 64, Vol. III—December 17th, 1887.

[Registered at the General Post Office as a Newspaper.]

Per Annum, 6s. 6d., post free.
Monthly Parts, 6d.; post free, 7½d.
Ditto per Ann., 7s. 6d. post free.

### THE HOSPITAL

Glasgow Western Infirmary — St Thomas's Hospital — Norfolk and Norwich Hospital

A WEEKLY INSTITUTIONAL JOURNAL OF

